# Complementary medicine use in stroke survivors: a US nationally representative survey

**DOI:** 10.1186/s12906-022-03525-0

**Published:** 2022-02-12

**Authors:** Wiebke K. Kohl-Heckl, Anna K. Koch, Holger Cramer

**Affiliations:** Department of Internal and Integrative Medicine, Evang. Kliniken Essen-Mitte, Faculty of Medicine, University of Duisburg-Essen, Essen, Germany

**Keywords:** Stroke survivors, Rehabilitation, Complementary medicine, Mind-body-medicine

## Abstract

**Background:**

Stroke is the second most common cause of death worldwide. Even after surviving, long-term rehabilitation often becomes necessary and does not always lead to complete recovery. Guidelines focus on prevention of risk factors and present concepts for rehabilitation after a stroke. Additional to these recommendations, complementary medicine (CM) utilization is common among patients with neurological conditions. CM also offers a wide range of therapies for both prevention and rehabilitation in stroke. There is limited information available on CM utilization among stroke survivors and differences to patients without former stroke diagnosis.

**Methods and results:**

This analysis was based on data of the 2017 National Health Interview survey (NHIS, *n* = 26,742; response rate 80,7%). We analyzed the prevalence of consultations among stroke patients with CM practitioners within the last 12 months and reasons for utilization. 3.1% of participants reported a stroke, individuals without a prior stroke diagnosis were more likely to have used CM in the past 12 months (31.3% without versus 28.9% with stroke). Consultations with a chiropractor and of using mind-body-medicine was higher in individuals without stroke diagnosis, while more stroke survivors had consulted a naturopath. Equal proportions had consulted a homeopath. Most common therapy approaches among stroke survivors were spiritual meditation (13.7%), progressive relaxation (5.4%), yoga (5.2%), mindfulness meditation (4.3%), mantra meditation (3.1%), guided imagery (2.6%) and tai chi (1.7%). CM use in stroke survivors was associated with female sex (adjusted odds ratio [AOR] = 2.12, 95% confidence interval [CI] = 1.56–2.88) and higher education (AOR = 1.94, CI = 1.42–2.65).

**Conclusion:**

Stroke patients were less likely to take advantage of complementary medicine than the general population. Since there are many safe and beneficial options, stroke survivors might profit from better information about the existing possibilities regarding prevention and rehabilitation.

## Introduction

According to the World Health Organization (WHO) worldwide about 17.9 million deaths were related to cardio- and cerebrovascular diseases in 2019 [1] and their rates, including strokes [2], are increasing. Right after acute myocardial attack, stroke – mostly ischemic – is the second most common cause of death worldwide [3] and fifth in the US [4]. Depending on the severity of destroyed brain tissue when suffering a stroke and a person’s age [5], stroke-related medical complications are numerous. Long-term rehabilitation might become necessary but can be complicated and does not always lead to a complete recovery. This might majorly worsen a patient’s outcome to reintegrate into a “normal” existence regarding all aspects of work and private life [5, 6]. All in all, the direct and indirect costs associated with stroke care majorly burden health care systems [7].

The same risk factors as in cardiovascular ones apply for the development of cerebrovascular diseases. According to the “Global Burden of Disease 2013” study around 90% of all risk factors are modifiable, whereas about 75% are associated with metabolic disorders and general behavioral choices [8]. Focusing on lifestyle changes and optimized treatment of risk factors, numerous guidelines (e.g. Eight Joint National Committee (JNC 8) [9], American Heart Association (AHA) [10] and many more) present guidelines based on multidimensional concepts to be most beneficial for patients. Additional utilization of complementary medicine (CM) might be an option to treat the prior mentioned risk factors as well [11–16] and overlaps with prevention measures [17]. After a prior event of cerebrovascular malfunction patients might utilize CM especially in secondary or tertiary prevention to improve their rehabilitation outcome. In general CM utilization is common in patients with stroke with 30.6% in 2007 [18], whereas 40% [19] of the general population did so in the same period of time. CM follows no strict definition but includes a lot of different categories such as mind-body-medicine (including e.g. meditation, mindfulness, relaxation techniques), traditional Chinese medicine (including e.g. herbal medicine and acupuncture), homeopathy, chelation therapy, osteopathy, chiropractic and many more. While prior research focused on individual aspects of CM (e.g. like mind-body therapies) among stroke patients [20] or analyzed their utilization among neurological diseases in general (including e.g. headaches, stroke, memory loss) [18], this is the first analysis comparing CM use only in stroke and non-stroke patients.

Our goal was to analyze the prevalence of CM utilization among adults with prior stroke diagnosis and differences to patients without. Furthermore, we studied varieties between categories of CM to characterize reasons for utilization among stroke survivors. While prior research focused e.g. on a general approach of complementary therapies of patients with neurological conditions or on the outcome of patients utilizing CM after stroke, this is the first analysis focusing only on stroke patients and their interest in complementary methods compared with non-stroke patients.

## Methods

### Study design

This cross-sectional analysis used data from the 2017 National Health Interview Survey (NHIS). Data, methods used in the analysis, and materials used to conduct the research are available online to any researcher for purposes of reproducing the results or replicating the procedure (https://www.cdc.gov/nchs/nhis/index.htm). All methods were performed in accordance with the relevant guidelines and regulations.

The NHIS is an annual nationally representative health survey of the non-institutionalized US population [21]. In 2017, the NHIS included 32,617 households and 26,742 adults provided data, indicating a response rate of 80.7% [22].

For this analysis, data from the NHIS Person File and NHIS Sample Adult File were used. A prior stroke diagnosis was queried as follows: “Have you ever been told by a doctor or other health professional that you had a stroke?” If this question was answered in the affirmative, the participant was considered a stroke survivor. Further, data on the socio-demographic characteristics age, sex, ethnicity, region, marital status, education, and employment as well as on CM use were included in the analysis. CM use was defined as i) having consulted with a chiropractor, a naturopath, a practitioner of chelation therapy, a practitioner of traditional medicine, and/or a homeopath in the past 12 months; and/or ii) having used mind-body medicine approaches (mantra meditation, mindfulness meditation, spiritual meditation, guided imagery, progressive relaxation, yoga, tai chi, and/or qi gong) in the past 12 months.

### Statistical analysis

The 12-month prevalence of any CM use was analyzed separately for individuals without a prior stroke diagnosis and for stroke survivors, as was the 12-month prevalence of consultations with a chiropractor, a naturopath, a practitioner of chelation therapy, a practitioner of traditional medicine, and/or a homeopath, and/or of the use of mind-body medicine approaches. Therapies used by less than 10 individuals in a specific cell were not further analyzed. Since the NHIS oversamples minorities, population-based estimates were calculated using weights calibrated to the 2010 census-based population estimates for age, gender, and ethnicity of the US civilian non-institutionalized population.

Sociodemographic and clinical characteristics were compared between a) individuals without a prior stroke diagnosis and stroke survivors using chi-squared tests, Fisher’s exact tests or unpaired t-tests as appropriate. Using the same models, sociodemographic and clinical characteristics were compared between stroke survivors who had used versus those who had not used CM in the past 12 months. The following independent variables were included in the analysis: age (linear variable), ethnicity (categories: non-Hispanic White, Hispanic, African American, Asian, other), region (categories: West, Northeast, Midwest, South), marital status (categories: not in relationship, in relationship), education (categories: less than college, some college or more), employment (categories: employed, unemployed), and health insurance status (categories: not insured, insured). To analyze independent predictors of CM use in the past 12 months, a backward stepwise multiple logistic regression analysis was utilized. In this analysis, only those predictors associated with mind-body medicine use in univariate analysis at a *p*-value of ≤0.10 were considered. Adjusted odds ratios with 95% confidence intervals and *P*-values were calculated using relative weights; and *P*-values ≤0.05 were considered statistically significant in regression analysis. The Statistical Package for Social Sciences (IBM SPSS Statistics for Windows, release 25.0. Armonk, NY: IBM Corp.) was used for all analyses.

## Results

A weighted total of 7,757,821 US adults (3.1%) had ever been diagnosed with stroke. Stroke survivors and individuals without a stroke diagnosis differed on all analyzed sociodemographic characteristics (Table 1).

**Table 1:** Comparison of characteristics in individuals without a stroke diagnosis and stroke survivors. Weighted data are representative for the civilian non-institutionalized population of the United States and do not reflect raw study numbers

Characteristics	No stroke diagnosis (weighted *n* = 238,899,450)	Stroke survivors (weighted *n* = 7,757,821)	*P* value
Age (Mean ± Standard deviation)	46.9 ± 18.0	65.5 ± 14.3	< 0.001
Sex			0.002
Male	114,828,688 (48.1%)	4,161,400 (53.6%)	
Female	124,070,762 (51.9%)	3,596,421 (46.4%)	
Ethnicity			< 0.036
Non-Hispanic White	16,866,8410 (70.6%)	5,209,325 (67.1%)	
Hispanic	28,450,939 (11.9%)	1,022,068 (13.2%)	
African American	26,154,790 (10.9%)	871,811 (11.2%)	
Asian	12,449,616 (5.2%)	470,530 (6.1%)	
Other	3,175,695 (1.3%)	184,087 (2.4%)	
Region			< 0.001
West	56,817,267 (23.8%)	1,560,205 (20.1%)	
Northeast	44,066,931 (18.4%)	1,102,129 (14.2%)	
Midwest	51,849,267 (21.7%)	1,943,502 (25.1%)	
South	86,165,985 (36.1%)	3,151,985 (40.6%)	
Education			< 0.001
Less than college	84,863,775 (35.7%)	3,719,522 (48.6%)	
Some college or more	153,129,584 (64.3%)	3,939,196 (51.4%)	
Employment			< 0.001
Not employed	86,137,494 (36.1%)	6,132,012 (79.0%)	
Employed	152,685,287 (63.9%)	1,625,809 (21.0%)	
Marital status			< 0.001
Not in a relationship	94,129,063 (39.5%)	3,671,551 (47.3%)	
In a relationship	144,393,462 (60.5%)	4,086,270 (52.7%)	
Health insurance			< 0.001
Not insured	25,484,799 (10.7%)	395,516 (5.1%)	
Insured	213,414,651 (89.3%)	7,362,305 (94.9%)	

Weighted frequencies or means are reported; P-values are derived from chi-squared tests, Fisher’s exact tests or unpaired t-tests using relative weights

Data Source: National Center for Health Statistics, National Health Interview Survey, 2017

**Fig. 1:**
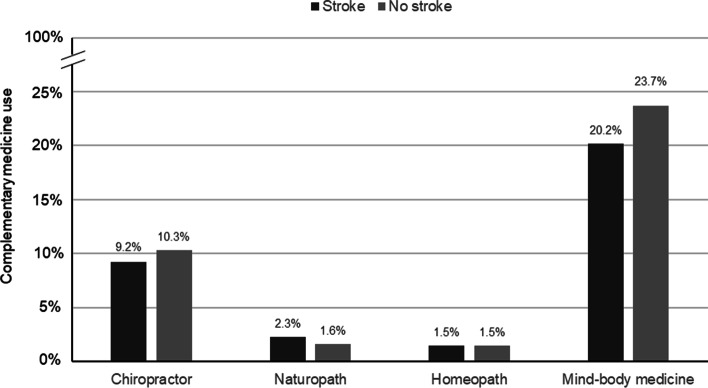
12-month prevalence of consultations with complementary medicine practitioners and of mind-body medicine use in stroke survivors and individuals without a stroke diagnosis. Weighted frequencies were used. Data Source: National Center for Health Statistics, National Health Interview Survey, 2017

Further, more individuals without a stroke diagnosis (weighted *n* = 74,718,521; 31.3%) than stroke survivors (weighted *n* = 2,242,068; 28.9%) had used CM in the past 12 months. Specifically, the 12-month prevalence of consultations with a chiropractor and of using mind-body medicine was higher in individuals without stroke diagnosis, while more stroke survivors had consulted a naturopath and equal proportions had consulted a homeopath (Fig. 1). Less than 10 stroke survivors had consulted with practitioners of traditional medicine or chelation therapy.

The most commonly used mind-body medicine modality in stroke survivors was spiritual meditation (13.7%) followed by progressive relaxation (5.4%), yoga (5.2%), mindfulness mediation (4.3%), mantra meditation (3.1%), guided imagery (2.6%), and tai chi (1.7%). Qi gong was used by less than 10 stroke survivors.

In both univariate analysis (Table 2) and multivariate logistic regression analysis (Table 3), CM use in stroke survivors was associated with female sex and higher education. Younger age and/or living in the US West or Midwest were associated with CM use in univariate analysis but were no independent predictors in multivariate analysis.

**Table 2:** Comparison of characteristics in stroke survivors using or not using complementary medicine. Weighted data are representative for the civilian non-institutionalized population of the United States and do not reflect raw study numbers

Characteristics	Not using complementary medicine (weighted *n* = 5,515,753)	Using complementary medicine (weighted n = 2,242,068)	*P*-value
Age (Mean ± Standard deviation)	66.4 ± 13.9	63.2 ± 15.0	0.003
Sex			< 0.001
Male	3,238,970 (58.7%)	922,430 (41.1%)	
Female	2,276,783 (41.3%)	1,319,638 (58.9%)	
Ethnicity			0.889
Non-Hispanic White	3,708,535 (67.2%)	1,500,790 (66.9%)	
Hispanic	733,847 (13.3%)	288,221 (12.9%)	
African American	588,816 (10.7%)	282,995 (12.6%)	
Asian	346,958 (6.3%)	123,572 (5.5%)	
Other	137,597 (2.5%)	46,490 (2.1%)	
Region			0.045
West	987,169 (17.9%)	573,036 (25.6%)	
Northeast	833,991 (15.1%)	268,138 (12.0%)	
Midwest	1,351,488 (24.5%)	592,014 (26.4%)	
South	2,343,105 (42.5%)	808,880 (36.1%)	
Education			< 0.001
Less than college	2,870,634 (53.0%)	848,888 (37.9%)	
Some college or more	2,549,795 (47.0%)	1,389,401 (62.1%)	
Employment			0.135
Not employed	4,435,507 (80.4%)	1,696,505 (75.7%)	
Employed	1,080,246 (19.6%)	545,563 (24.3%)	
Marital status			0.110
Not in a relationship	2,710,014 (49.1%)	961,537 (42.9%)	
In a relationship	2,805,739 (50.9%)	1,280,531 (57.1%)	
Health insurance			0.491
Not insured	262,574 (4.8%)	132,942 (5.9%)	
Insured	5,253,179 (95.2%)	2,109,126 (94.1%)	

Weighted frequencies or means are reported; P-values are derived from chi-squared tests, Fisher’s exact tests or unpaired t-tests using relative weights

Data Source: National Center for Health Statistics, National Health Interview Survey, 2017

**Table 3:** Individual predictors of complementary medicine use in stroke survivors

Predictor	Adjusted odds ratio (95% confidence interval)	*P*-value
Female sex	2.12 (1.56–2.88)	< 0.001
Education (some college or more)	1.94 (1.42–2.65)	< 0.001

*P*-values are derived from logistic regression analysis using relative weights

Data Source: National Center for Health Statistics, National Health Interview Survey, 2017

## Discussion

This study reports the use of complementary medicine among stroke survivors and non-stroke patients. In general, stroke survivors were slightly less likely to consult with a CM practitioner or to use mind-body medicine than the general population.

In our analysis, 3.1% of participants had been diagnosed with a stroke. This is an increase compared with data from NHIS 2002 (2.6% of participants indicated a stroke [23]), NHIS 2007 (0.5% of participants were labeled as stroke patients [18]), or other countries, like Canada (1.15% in 2015 [24]).

Overall, the utilization of complementary therapies both with (28.9%) or without (31.3%) a stroke diagnosis is comparable with a general complementary health approach regarding previous data [19]. It has to be pointed out, that we do not know whether participants with a prior stroke diagnosis utilized various CM for stroke-related reasons, as the NHIS does not include this in their 2017 questionnaire. Therefore, it can only be hypothesized why these therapies might have been utilized by patients. It is possible, that the utilization may be for general well-being without being stroke-related. As well, we do not know whether people with a stroke diagnosis used these prior mentioned CM therapies instead or additionally to conventional medicine. This is important to highlight as CM should not replace but support conventional therapy in integrative concepts. Conventional therapies like medication and regular check-ups have to stay the base of therapeutic concepts.

So far, there is no prior research comparing non-stroke patients with stroke survivors, but data are available for different neurological conditions. In comparison to other neurological conditions like memory loss, migraine, or seizures, stroke patients showed a lower rate of CM utilization (30.6%) [18].

Reasons for a lower approach in stroke patients seem various. While there are many complementary therapies available that can possibly improve a patients´ outcome during stroke recovery, some others are critically discussed.

While on one hand chiropractic therapy is a treatment option for muscular pain – also after a stroke –, especially neck manipulation is still considered to be done with caution. For years there have been worries that cervical manipulative therapy, which can be part of chiropractic treatment, might lead to arterial neck dissections and stroke [25]. After only focusing mostly on case reports, an article from 2010 summarized available data on cervical manipulative therapy and stroke. This research indicated that visiting a chiropractor seems not to be a cause of vertebral artery dissection or stroke. Unspecific warning signs like headaches or neck pain can be related to a stroke and may be the reason for patients consulting a chiropractor in the first place. However, suffering from a diagnosed stroke some time after such an intervention seems independent to the manipulation itself but only time-related. With this knowledge it may be possible for chiropractors to diagnose stroke-suspicious symptoms and intervene early [26]. Furthermore, chiropractic therapy may be included as an additional and safe treatment option in rehabilitation after stroke.

Regarding the therapies which were most commonly used, our presented population of stroke survivors most frequently used spiritual meditation among available mind-body-medicine therapies, followed by progressive relaxation and yoga. Since the majority of risk factors apply for both cardiovascular and cerebrovascular diseases, conventional therapeutic recommendations like lifestyle modification are similar. Furthermore, the American Heart Association published a scientific statement in 2017 regarding meditation as an additional treatment option for cardiovascular diseases and their risk factors. Considering the little effort meditation takes (low costs, easy access, low risk) it can be considered as a reasonable addition to a guideline-based therapy for both diseases and risk factors [27]. Furthermore, especially transcendental meditation seems to be useful to lower an elevated blood pressure, while there is no significant evidence for the benefit of progressive relaxation [28]. Yoga also seems to reduce common physical risk factors for cardiovascular and cerebrovascular disease such as hypertension, obesity and hyperlipidemia [29].

In addition to motor disability, speech/language deficits and cognitive dysfunction, an estimated one third of stroke survivors suffer from depression. Likewise, around the same amount is burdened with anxiety and post-traumatic stress disorder [30, 31]. Regarding stroke in general, a Cochrane review summarizes the rather sparse data on yoga as a rehabilitation therapy after stroke. In conclusion, yoga has the potential to be accepted as part of patient-focused stroke rehabilitation [32]. Regarding psychological comorbidities in general, yoga for example has been shown to have positive effects on depression [33]. The same applies for meditation, especially mindfulness meditation [34]. As further mentioned for complementary therapies, also yoga and meditation are practiced more often by younger, female persons [35, 36]. Therefore, it is not surprising that the older and more male stroke patients are less likely to practice MBM of which yoga and meditation are part of. Because of the potentially positive effects, it would make sense to inform and motivate other groups in this regard and to include such complementary therapies in stroke rehabilitation programs.

Considering other reasons for a lower prevalence of CM use our research shows that people with stroke are significantly older than the general population. Among stroke patients, CM utilization was more common when patients were female and of younger age. This observation is in line with prior research findings: also in cardiovascular disease, complementary health care utilization is – among other factors -associated with female gender.

Another reason for a lower utilization among participants with a prior stroke diagnosis may be based on the disease and its sequela itself. People may suffer from lack of physical and/or cognitive dysfunction that complicates their access to various CM therapies. The survey does not cover information regarding these specific reasons.

As an annual cross-sectioned survey, these presented results have limitations. Our data showed a negative relationship between utilization of complementary healthcare and a prior stroke diagnosis. As this survey contains self-reported data from non-institutionalized US citizens, no cross-validation from medical records was available. Since a stroke survivor was only defined by questioning patients themselves, there is a chance prior diagnoses were misunderstood or completely missed/forgotten by participants. Also, the 2017 NHIS survey only assessed a limited content of complementary therapies. Acupuncture or use of supplements were not assessed in the 2017 survey, though both present highly utilized therapies in general. It would be beneficial to focus on a more detailed collection of information in future studies.

## Conclusion

The present study analyses population-based estimates regarding CM use in US stroke survivors. Although the majority of stroke survivors still have to deal with the consequences months and sometimes years after a stroke and CM offers a wide range of rehabilitation options, a smaller proportion of them takes advantage of complementary therapy approaches compared to the population without stroke. Younger women suffering from a prior stroke were most likely to utilize CM. Since there are many safe and beneficial options, the respective target groups might profit from better information about the existing possibilities.

## Data Availability

For this analysis, data from the NHIS Person File and NHIS Sample Adult File were used. Data, methods used in the analysis, and materials used to conduct the research are available online to any researcher for purposes of reproducing the results or replicating the procedure (https://www.cdc.gov/nchs/nhis/index.htm).

## Abbreviations

CM: Complementary medicine
NHIS: National Health Interview Survey
WHO: World Health Organization
